# Supplementary material for: Estimating the Associated Burden of Illness and Healthcare Utilization of Newly Diagnosed Patients Aged ≥65 with Mantle Cell Lymphoma (MCL) in Ontario, Canada
Source: Curr Oncol. 2023 Jun 8;30(6):5529–45. doi: 10.3390/curroncol30060418 (PMC10297405; doi:10.3390/curroncol30060418)
Supplement: Supplementary file 1 [file curroncol-30-00418-s001.zip › curroncol-2298938-supplementary.pdf]

SUPPLEMENTAL MATERIALS

Supplementary Table S1. Regimens considered as first-line therapy for MCL

Abbreviated regimen name	Full regimen name
BR	Bendamustine, rituximab [Rituxan]
CHOP	Cyclophosphamide, doxorubicin, vincristine, prednisone
R-CHOP	Rituximab, cyclophosphamide, doxorubicin [Hydroxydaunorubicin], vincristine [Oncovin], prednisone
R-DHAP*	Rituximab, dexamethasone, high-dose cytarabine (Ara-C), cisplatin
CVP	Cyclophosphamide, vincristine, prednisone
R-CVP	Rituximab, cyclophosphamide, vincristine, prednisone
Hyper-CVAD	Cyclophosphamide, vincristine, doxorubicin [Adriamycin], dexamethasone, methotrexate, cytarabine
R-HyperCVAD*	Rituximab, cyclophosphamide, vincristine, doxorubicin, dexamethasone, methotrexate, cytarabine
CHLO	Chlorambucil
CHLO-R	Chlorambucil, rituximab
CYCL(PO)	Cyclophosphamide (oral)
FC	Fludarabine, cyclophosphamide
FC-R	Fludarabine, cyclophosphamide, rituximab
FR	Fludarabine, rituximab
FLUD-CYCLO-MITOXAN	Fludarabine, cyclophosphamide, mitoxan
VR-CAP*	Bortezomib, rituximab, cyclophosphamide, doxorubicin, prednisone
LR*	Lenalidomide, rituximab
RBAC500*	Rituximab, bendamustine, cytarabine
Nordic regimen*	Dose-intensified induction with rituximab plus cyclophosphamide, vincristine, doxorubicin, prednisone alternating with rituximab plus high-dose cytarabine

*Regimens not identified in the MCL cohort but listed as recommended 1st LoT for MCL in guidelines

Supplemental Table S2. Patient selection into MCL cohort, Ontario, Canada, 2013 – 2016

Cohort Selection Criteria		Remaining Patients		Excluded patients
		No.	%	
MCL cohort inclusion criteria	Note			
Diagnosis prior to December 31st, 2016 with MCL (Morphology code = 96733)		*2,100*		
Exclusion Criteria				
Invalid OHIP card number and age<65		*1,270*		
Invalid or incomplete records (missing age, sex, LHIN, income quintile, and rurality, or death before MCL diagnosis)		*1,225*		
Age ≥105 years at MCL diagnosis		*1,225*		
Non-Ontario resident		*1,225*		
Undetermined MCL diagnosis (cancers other than MCL diagnoses on the same date)		*1,212*		
No OHIP eligibility at diagnosis		*1,206*		
Death date recorded on the date of index diagnosis		*1,206*		
Excluding those having MCL diagnosis date before January 1, 2013		*325*		
Excluding those having SCT any time before the end of the Analysis Period (including)		*313*		
**Pool of MCL Patients**		***313***	100.0%	
MCL ≥ 65 in 1LoT subgroup inclusion criteria		*313*		
Patients in the MCL≥ 65 cohort who has record in ALR within 3 years from index date and up to 31Dec2019		*213*	68.1%	*100*
Excluding patients who participated in a clinical trial	(n=*1-5)			**1-5*
Patients received confirmed 1st LoT	Excluded patients including those who participated in a trial	*183*	58.5%	*30*
Excluding patients who had refractory disease or treatment intolerance		*161*	51.4%	*22*
**MCL cohort**		***161***		
Matching to 4 general population controls		*159*	50.8%	*2*
**Matched MCL Patients**		***159***		

*Values were suppressed as per ICES reporting standards to reduce the risk of patient re-identification

Supplemental Table S3. First line therapy received by the MCL cohort^

Lines of Therapy		n (%)
Regimen category	**Overall**	**161^ (100%)**
	BR	125 (78.1%)
	R-CHOP	21 (13.1%)
	CHOP	0
	R-CVP	*9-13
	CVP	0
	Hyper-CVAD	0
	CHLO	*1-5
	CHLO-R	0
	CYCL(PO)	0
	FC	0
	FC-R	*1-5
	FR	0
	FLUD-CYCLO-MITOXAN	0
R-maintenance for 1st LoT, n (%)		126 (78.3%)
Time to next therapy (TTNT), days	n (%)	38 (23.6%)
(from initiation of 1st LoT to the initiation of 2nd LoT)	Mean, SD	465.6, 220.6
	Median (IQR)	462.5 (297-628)
	Min - Max	29-988

^MCL cohort prior to general population matching

*Double suppression was conducted according to ICES reporting standards to reduce the risk of patient re-identification

Supplemental Table S4. Demographic and clinical characteristics for matched MCL patients, by LoT categories

Demographic and Clinical Characteristics		BR (n=123)	Other (n=36)
Sex			
	Female	35 (28.5%)	16 (44.4%)
	Male	88 (71.5%)	20 (55.6%)
Age			
	Mean, SD	75.0 ± 6.0	76.1 ± 6.7
	Median (IQR)	74 (70 - 80)	76 (72 - 81)
	Min - Max	65 – 92	65 – 91
	65 - 74 years	63 (51.2%)	14 (38.9%)
	75+ years	60 (48.8%)	22 (61.1%)
Rural residence			
	Urban	*100 - 104	*31 - 35
	Rural	*19 - 23	*1 - 5
Income quintile			
	Q1, lowest	*19 - 23	*1 – 5
	Q2	*26 - 30	*3 – 7
	Q3	23 (18.7%)	6 (16.7%)
	Q4	23 (18.7%)	10 (27.8%)
	Q5, highest	29 (23.6%)	11 (30.6%)
New Ontario Resident at diagnosis		*1 - 5	*1 – 5
Ontario Health Region			
	Central	40 (32.5%)	17 (47.2%)
	East	*31 - 35	*7 – 11

	West	36 (29.3%)	7 (19.4%)
	North	*12 - 16	*1 - 5
Year of MCL cancer diagnosis			
	2013	*14 – 18	*16 – 20
	2014	33 (26.8%)	8 (22.2%)
	2015	35 (28.5%)	7 (19.4%)
	2016	*37 - 41	*1 - 5
Cancer stage at diagnosis			
	Stage I	0 (0%)	*1 – 5
	Stage II	*1 – 5	0 (0%)
	Stage III	*16 – 20	*1 – 5
	Stage IV	*25 – 29	*4 – 8
	Missing	74 (60.2%)	26 (72.2%)
Cancer history (assessed within 5-year lookback)^		*10 - 14	*1 - 5
Charlson's co-morbidity index (assessed within 2-year lookback)			
	0	*18 - 22	*1 – 5
	1	*1 – 5	*1 – 5
	2	*1 – 5	*1 – 5
	3+	*1 – 5	*1 – 5
	Missing	93 (75.6%)	26 (72.2%)
Comorbidities, any time before index date (ICES-derived cohorts)			
	Diabetes mellitus	28 (22.8%)	10 (27.8%)

	Congestive heart failure (CHF)	*3 – 7	*1 – 5
	Chronic obstructive pulmonary disease (COPD)	29 (23.6%)	6 (16.7%)
	Rheumatoid arthritis (RA)	*1 - 5	*1 – 5
	Crohn's disease / Ulcerative colitis	*1 - 5	0 (0%)
Time from index date to end of follow-up			
	Mean, SD	947.2 ± 296.9	806.4 ± 379.0
	Median (IQR)	1,095 (1,025 - 1,095)	1,095 (503 - 1,095)
	Min - Max	28 - 1,095	45 - 1,095

^Due to small cell sizes, the types of previous cancers could not be reported

*Double suppression was conducted according to ICES reporting standards to reduce the risk of patient re-identification
